# An exploratory analysis of work engagement among women with and without disordered eating

**DOI:** 10.1186/s12905-021-01429-8

**Published:** 2021-08-18

**Authors:** Mikaela Willmer, Josefin Westerberg Jacobson, Magnus Lindberg

**Affiliations:** 1 Department of Caring Sciences, University of Gävle, 801 76 Gävle, Sweden; 2 Department of Public Health and Caring Sciences, University of Uppsala, Uppsala, Sweden

**Keywords:** Disordered eating, Eating behavior, Occupational health, Work engagement, Workplace health

## Abstract

**Background:**

Around 10% of the female population is estimated to have a subclinical eating disorder. Many of these women are of working age. Previous research has shown associations between unhealthy eating behaviors and occupational stress or burnout. However, no previous study has explored the association between disordered eating and work engagement, a positive, fulfilling, affective-cognitive state of mind which has been positioned as the conceptual opposite of burnout. Thus, that was the aim of the present study.

**Methods:**

In this cross-sectional study, a sample of 701 Swedish women completed the 9-item Utrecht Work Engagement Scale (UWES-9) and the Eating Disorder Examination Questionnaire (EDE-Q). They were divided into a Healthy Eating (HE) and a Disordered Eating (DE) group based on their EDE-Q scores. The Mann–Whitney U test was used to test the association between disordered eating and work engagement. The Kruskal Wallis test was used to assess the associations between educational level, marital status and age group, and work engagement.

**Results:**

Neither the UWES scores nor the EDE-Q scores were found to have a normal distribution. Non-parametric testing showed that the DE group reported significantly lower work engagement than the HE group (p = 0.016). There were no significant associations between education, marital status or age (independent variables) and work engagement (dependent variable) (p = 0.826, 0.309, and 0.349, respectively).

**Conclusion:**

These findings indicate that work engagement may play a role in disordered eating, and that there is a need for future research to consider the workplace environment as a potential source for altering disordered eating behaviors.

## Background

Food provides energy to the body, and energy is vital for an individual's mental health and work performance. Disordered eating is associated with mental consequences and refers to disturbed or unhealthy weight control thoughts and behaviors. Skipping meals, using food supplements and substitutes, feeling a loss of control while eating, using diet pills, self-induced vomiting, binge eating, viewing exercise as a means of earning food, excessive exercise, and overvaluation of one’s weight and shape are all thoughts and behaviors that may increase in severity and frequency to become full-blown eating disorders [1].

The prevalence of diagnosed eating disorders in the general population has been estimated to about 1–4% [2]. In the working population, an eating disorder is most likely to be found in the 16–30 year age range. This means that there is a significant number of individuals in the workplace who are experiencing disordered eating behaviors in their everyday lives. Additionally, the prevalence of subclinical disordered eating behaviors is of interest. The prevalence of subclinical eating disorders depends, naturally, on how they are defined. At least two previous studies [3, 4] used a 5-item screening tool (SCOFF), and defined any respondents who answered”yes” to 2 or more items as having a subclinical eating disorder. Beals [5] used five different self-report eating disorder pathology questionnaires, and defined those who scored above the cut-off on at least three of these as having a subclinical eating disorder. Völker et al. [6] chose to categorize those who answered”yes” to any one of a number of different questions regarding frequency of eating disorder-related behaviors, such as restrictive eating, binge eating or compensatory purging. Hence, there is no consensus on the definition of subclinical eating disorders. Two studies of the prevalence of subclinical eating disorders in the general female population have found it to be 9% and 12%, respectively [3, 4].

Having employment is typically associated with positive mental health outcomes. The workplace has, however, been shown to be a potentially difficult site for symptom management for women with eating disorders. Certain workplace stressors such as leader intolerance, co-worker awareness, schedule conflicts, and health-focused workplaces moderate the type of strategy used for stress management, and the findings of Siegel and Sawyer [7] indicate that management techniques used may contribute to either relapses or recovery of the eating disorder.

Previous studies have investigated the associations between eating behavior and job stress, which in its most severe and chronic form may lead to occupational burnout, a condition characterised by exhaustion, cynicism towards one’s job, and inefficacy [8]. King et al. [9] surveyed 435 nurses, and found an association between job stress and disordered eating. In addition to this, a study by Nevanperä et al. [10], exploring the associations between burnout and eating behavior in Finnish employees, found a significant association between symptoms of burnout and higher self-reported emotional and uncontrolled eating. Along similar lines, a review study found associations between life stress, daily hassles and disordered eating, although uncertainties remain regarding the causality and directions of these associations, since the included studies were mainly either cross-sectional or retrospective, meaning that recall bias may have affected the findings [11].

In light of the above, it seems like the available literature indicates that a negative work environment, characterized by job stress and even burnout, has at least an association with disturbed eating behavior, although research in the area is sparse. This leads to the question of whether there may also be an association between disturbed eating and work engagement. Work engagement is defined as a positive, fulfilling, affective-cognitive state of mind and has been positioned as the conceptual opposite of burnout [12, 13]. Schaufeli and colleagues have suggested that three sub-dimensions may be used to capture this state: vigor, dedication and absorption [12, 14]. Vigor refers to level of energy at work, willingness to invest effort in one’s work, the ability to keep working without experiencing fatigue, and displaying mental resilience at work. Dedication is shown through feelings of enthusiasm, pride, inspiration, and positive challenge. Finally, the state of absorption refers to concentration, devotion and getting carried away by one´s work [12, 14, 15].

Previous studies have shown a correlation between higher work engagement and better mental and physical health [16, 17]. An employee’s level of work engagement is known to be influenced by individual factors such as education level, but also by work-related and contextual factors [18]. Furthermore, it also depends on which country the person works in. The most engaged workers within the European Union are found in the Netherlands, Ireland, and Belgium. Swedish workers are moderately engaged, and the least engaged workers are located in the Balkan [19]. European women are significantly more engaged at work than men, and there is a clear linear trend between higher education level and higher total UWES scores [18]. An association between work engagement, job satisfaction and quality of life has been shown in previous research [20]. However, the authors of the present study have failed to find any previous research focusing on a possible association between work engagement and disordered eating. Investigating such an association might have implications for understanding the importance of the workplace as an arena for eating disorder recovery, a hitherto sparsely researched area, and may also inform clinicians on how the workplace environment may influence the eating behavior of those with disordered eating. Thus, the aim of the present study was to explore associations between disordered eating and work engagement in a sample of Swedish women.

## Methods

### Participants

The sample was comprised of 26–36-year-old women originating from the general population cohort who participated in the 20-year follow-up of the Swedish longitudinal cohort study of investigation of dieting behaviors in adolescent girls (IDA) [21, 22]. Only women who self-reported that they were currently employed were included in the current study. Women who reported that they were on parental leave at the time of data collection were excluded. The final sample consisted of 701 women.

### Measures and procedure

Following ethical approval granted by the Regional Ethical Review Board in Uppsala (reg no:2014/401), a cross-sectional survey was conducted between March and September 2015. The questionnaires were distributed by regular mail, and returned in a prepaid envelope or through an online survey system, by the respondents’ own choice. One postcard reminder was sent; the overall study response rate was 69%.

Work engagement was assessed with the commonly used 9-item Utrecht Work Engagement Scale (UWES-9). The items of the UWES-9 are scored on a 7-point Likert scale from 0 to 6, with 0 representing Never and 6 representing Always. The higher each item was rated, the higher the overall work engagement [15]. The UWES score is calculated as a mean value of the responses given to the 9 items. Based on norm scores, the UWES-9 total score may be transformed into five categories of work engagement; Very low < 1.77, Low 1.78–2.88, Average 2.89–4.66, High 4.67–5.50, or Very high > 5.51 [12]. In national representative samples across Europe, the mean score on UWES in Europe was 3.94, and for Sweden 3.92 [19].

The UWES-9 was originally designed with three subscales, measuring Vigor, Dedication and Absorption, respectively. However, there have been doubts about the factorial validity of this structure [23]. Consequently, the authors conducted exploratory and confirmatory factor analysis of the sample used in the current study, and found that a one-factor structure best fit the data [24]. Disordered eating was assessed with the Eating Disorder Examination Questionnaire version 6.0 (EDE-Q) [25]. The EDE-Q is a 28-item self-report measure adapted from the EDE [26]. It provides a global scale as well as four subscales; the global scale is the average of the subscale scores. The global mean value of the EDE-Q is calculated by summing the four subscales scores and dividing the total by four. Its theoretical range is 0–6 [25]. The subscales are: Eating concern (preoccupation with thoughts about food and eating, feelings of anxiety and guilt after eating), Shape concern (preoccupation with thoughts about body shape and dissatisfaction about one’s own body), Weight concern (preoccupation with thoughts about weight, wish to lose weight) and finally Restraint (the extent of restrictive eating, amounts of food and nutrient content). The EDE-Q focuses on the past 28 days and measures the core pathology of eating disorders. The EDE-Q is scored on a 7-point Likert scale, except for the items relating to the frequency behaviors, which are assessed in terms of the number of episodes occurring during the past 28 days. Higher scores are indicative of higher eating pathology. A community sample of 243 young women yielded a mean global score of 1.404 [27].

Educational level was divided into three groups, with “Low” being 12 years of mandatory and upper secondary education, “Medium” being less than three years of higher education, and “High” being at least three years of university or comparable higher education.

Marital status was divided into single/divorced/in a relationship but not cohabiting, or married/cohabiting.

### Statistical analysis

All statistical analyses were performed using Stata version 14 [28] and a p-value of < 0.05 was considered statistically significant. Descriptive statistics, including frequencies, means and standard deviations, were used to summarize the sample demographics and self-rated questionnaire responses.

Following the recommendation by Fairburn and Beglin [25], missing values in the EDE-Q subscale items were not replaced. As long as a minimum of half of the items in the subscale had been rated, we calculated the EDE-Q subscales by adding together the relevant available items for each subscale and then divided the sum by the number of rated items. The sample was divided into a “healthy eating” (HE) group and a “disordered eating” (DE) group, based on the EDE-Q scores. In previous work to determine Swedish population norms for EDE-Q scores of young women, those whose EDE-Q scores were at least two standard deviations above the sample mean have been considered to have a clinical eating disorder [29]. However, as the current study aimed to identify those with subclinical eating disorders, the decision was made to place those whose score was at least one standard deviation above the sample mean in the DE group. The Shapiro–Wilk test was used to estimate the normality of the UWES and EDE-Q scores for the sample [30]. A one-sample t-test was used to compare the sample mean with the mean previously seen for Swedish workers [19]. The Mann–Whitney U test was used to test the association between disordered eating and work engagement [31]. Finally, in order to determine whether there was an association between educational level, marital status or age group and work engagement in our sample, a Kruskal–Wallis test was employed [32].

## Results

Table 1 shows some background characteristics of the participants. The majority of the sample (60%) had at least three years of higher education, and 75% were married or cohabiting.

**Table 1:** Demographic characteristics of the participants

N = 701	Mean (SD) or freq (%)
Age (years)	31.8 (2.9)
Marital status	
Single, divorced or not living w partner	168 (24.0%)
Married/cohabiting	529 (75.5%)
Education	
Low	136 (19.4%)
Medium	141 (20.1%)
High	424 (60.5%)

As described above, the sample was divided into a “healthy eating” (HE) group and a “disordered eating” (DE) group, based on the EDE-Q scores. The DE group comprised 123 individuals, or 17.5% of the entire sample. Table 2 shows the UWES and EDE-Q scores for the entire sample as well as for the two groups separately.

**Table 2:** Mean EDE-Q and UWES scores for the entire sample and for the “disordered eating” and “healthy eating” groups

	Entire sample (n = 701, 100%)	HE group (n = 578, 82.4%)	DE group (n = 123, 17.5%)
Mean score (SD) UWES	4.06 (1.18)	4.13 (1.17)	3.76 (1.40)
Range UWES	0–6	0–6	0–6
Median UWES	4.33	4.39	4.11
Mean score EDE-Q (SD)	1.21 (1.07)	0.83 (0.66)	3.02 (0.67)
Range EDE-Q	0–5.58	0–2.26	2.28–5.58
Median EDE-Q	0.89	0.64	2.86

*SD* standard deviation, *UWES* Utrecht Work Engagement Scale, *EDE-Q* Eating Disorder Examination Questionnaire, *HE* healthy eating, *DE* disordered eating

The one-sample t-test comparing the sample mean UWES score (4.06) to the mean score (3.92) of a representative national Swedish sample showed a statistically significant difference (p = 0.0016).

When assessing normality using the Shapiro–Wilk test, it was found that neither the UWES scores nor the EDE-Q scores was normally distributed (UWES: W = 0.946; p < 0.0001; EDE-Q: W = 0.904; p < 0.0001). Hence, the decision was made to proceed with a non-parametric test to assess the difference in UWES scores between the HE and DE groups. The results of a Mann–Whitney U test showed that the participants in the DE group had significantly lower work engagement, as measured by UWES (p = 0.016).

In order to investigate whether education level, marital status or age was also associated with work engagement, we conducted Kruskal–Wallis tests with UWES score as the dependent variable and educational level, marital status or age (divided into two groups with 26–30-year-olds in one group and 31–36-year-olds in the other) as the independent variable. These tests showed no significant differences in work engagement (p = 0.826, 0.309, and 0.349, respectively).

## Discussion

The results of the present study showed a significant association between self-reported disordered eating behavior and lower work engagement. Education, marital status, or age was not shown to be associated with work engagement. The mean UWES score for the entire sample was 4.06, significantly higher than a previous study, which showed a mean score of 3.92 [19]. However, both these mean scores fall within the category of “average work engagement” [12].

As for the EDE-Q score, the sample mean was found to be 1.21, slightly but not remarkably lower than a previously reported community sample mean of 1.40 [27].

Work engagement is a predictor of both physical and mental well-being, and is one important construct of adults’ happiness and well-being at work [13]. As the workplace is one of the locations where most adults spend much of their time, it is an important arena for the promotion of well-being and health. Although there is a lack of research regarding the relationship between disordered eating and work engagement, the results of the present study may be put into context with some earlier work. King et al. [9] have shown a relationship between job stress and disordered eating. As job stress (and in its most serious form, burnout) is seen as the conceptual opposite of work engagement, it is an interesting finding that our results seem to point in the same direction as those in the study by King. Taken together, this may indicate that higher levels of stress and less work engagement seem to have an association with disordered eating behavior.

In addition to the above, a Dutch study on slightly older workers found that work engagement could predict mental health, with higher UWES scores at baseline being associated with better mental health a year later [33]. This may be of interest in relation to the results of the present study, as interventions to improve work engagement or occupational health may also improve the mental health of those affected by disordered eating. Of course, given the cross-sectional nature of this study, it is not possible to draw any conclusions about causality, based on its results. It is possible that disordered eating behavior, with its effects on mental and physical health, causes the individual to experience lower work engagement. It is also possible that individuals use disordered eating behavior as a way to cope with stress and low work engagement. A review study by Ball and Lee [11] did indeed find associations between life stress, daily hassles and disordered eating, strengthening this hypothesis. Most of the studies included in that review were either retrospective, meaning that there was a risk of bias, or cross-sectional, meaning that it is not possible to draw conclusions about causality.

Educational level was not found to be associated with work engagement in the present study. Although some previous studies have found an association, our results are in agreement with studies on Iranian hospital staff and Turkish bank staff, which failed to show an association between educational level and work engagement [34, 35].

There are very few studies investigating the role of the workplace for eating disorder recovery. A qualitative study by Siegel and Sawyer [7] showed that the participants placed great importance on workplace factors, such as supportive managers and colleagues, when it came to either reach or stay in recovery. Previous research has also shown that the workplace is an important arena for recovery from other types of mental ill-health, such as depression and anxiety [36]. Taken together with evidence that interventions in the workplace also have the potential to improve people’s eating habits and promote health [37], it seems like the possibility of workplace interventions to help people recover from disordered eating may be an avenue of research worth exploring.

### Strengths and limitations

The present study has strengths, as well as limitations. The large sample size of 701 women and the high response rate (69%) for the data collection questionnaires must be considered strengths. However, the participants are the remainder of a larger cohort, recruited in 1995, as described in the Methods section, meaning that there has likely been some selection regarding who has remained in the study and who has dropped out. Indeed, 60% of the sample reported having a university education, which is much higher than the comparable section of the Swedish population, where 35% of women between the ages of 24 and 35 have university degree [38].

On the subject of generalizability, the sample only includes women, meaning that the results may not be applicable to men. It is rarer for men to suffer from disturbed eating, although this is likely to be partly due to underreporting [39]. Nonetheless, future research should take possible gender differences into account.

## Conclusion

The results of the current study showed an association between disordered eating and lower work engagement, whilst educational level, marital status or age group were not shown to have an association with work engagement. These findings indicate that work engagement may play a role in disordered eating, and that there is a need for future research to consider the workplace environment as a potential source for altering disordered eating behaviors.

## Data Availability

The datasets generated and/or analysed during the current study are not publicly available due to the fact that participants of this study did not agree for their data to be shared publicly, and it is not included in the ethical review board permission, but are available from the corresponding author on reasonable request.

## Abbreviations

DE: Disordered Eating
EDE-Q: Eating Disorder Examination Questionnaire
HE: Healthy Eating
IDA: Investigation of Dieting behaviors in Adolescent girls
UWES: Utrecht Work Engagement Scale
